# The change of gut microbiota‐derived short‐chain fatty acids in diabetic kidney disease

**DOI:** 10.1002/jcla.24062

**Published:** 2021-10-24

**Authors:** Chenyu Zhong, Zhiwei Dai, Lingxiong Chai, Lingping Wu, Jianhui Li, Weiying Guo, Jie Zhang, Qun Zhang, Congping Xue, Haixue Lin, Qun Luo, Kedan Cai

**Affiliations:** ^1^ Department of Nephrology HwaMei Hospital University of Chinese Academy of Sciences; Ningbo Institute of Life and Health Industry University of Chinese Academy of Sciences Ningbo China; ^2^ Department of Endocrinology HwaMei Hospital University of Chinese Academy of Sciences; Ningbo Institute of Life and Health Industry University of Chinese Academy of Sciences Ningbo China

**Keywords:** acetate, butyrate, diabetes mellitus, diabetic kidney disease, gastrointestinal microbiome, propionate, short‐chain fatty acids

## Abstract

**Background:**

Previous studies found the dysbiosis of intestinal microbiota in diabetic kidney disease (DKD), especially the decreased SCFA‐producing bacteria. We aimed to investigate the concentration of the stool and serum short‐chain fatty acids (SCFAs), gut microbiota‐derived metabolites, in individuals with DKD and reveal the correlations between SCFAs and renal function.

**Methods:**

A total of 30 participants with DKD, 30 participants with type 2 diabetes mellitus (DM), and 30 normal controls (NC) in HwaMei Hospital were recruited from 1/1/2018 to 12/31/2019. Participants with DKD were divided into low estimated glomerular filtration rate (eGFR)(eGFR<60ml/min, n=14) and high eGFR (eGFR≥60ml/min, n=16) subgroups. Stool and serum were measured for SCFAs with gas chromatograph‐mass spectrometry.

**Results:**

The DKD group showed markedly lower levels of fecal acetate, propionate, and butyrate versus NC (*p*<0.001, *p*<0.001, *p*=0.018, respectively) [1027.32(784.21–1357.90)]vs[2064.59(1561.82–2637.44)]μg/g,[929.53(493.65–1344.26)]vs[1684.57(1110.54–2324.69)]μg/g,[851.39(409.57–1611.65)] vs[1440.74(1004.15–2594.73)]μg/g, respectively, and the lowest fecal total SCFAs concentration among the groups. DKD group also had a lower serum caproate concentration than that with diabetes (*p*=0.020)[0.57(0.47–0.61)]vs[0.65(0.53–0.79)]μmol/L. In the univariate regression analysis, fecal and serum acetate correlated with eGFR (OR=1.013, *p*=0.072; OR=1.017, *p*=0.032). The correlation between serum total SCFAs and eGFR showed statistical significance (OR=1.019, *p*=0.024) unadjusted and a borderline significance (OR=1.024, *p*=0.063) when adjusted for Hb and LDL. The decrease in serum acetate and total SCFAs were found of borderline significant difference in both subgroups (*p*=0.055, *p*=0.050).

**Conclusion:**

This study provides evidence that in individuals with DKD, serum and fecal SCFAs levels (fecal level in particular) were lowered, and there was a negative correlation between SCFAs and renal function.

## Graphical abstract

In this study,we measured the concentrations of fecal and serum SCFAs in the participants with DKD and it showed that serum and fecal SCFAs levels (fecal level in particular) were lowered, and there was a negative correlation between SCFAs and renal function.

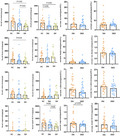

## 1 INTRODUCTION

Diabetic kidney disease (DKD) is the most serious complication of diabetic mellitus (DM) and the leading cause of chronic kidney disease (CKD) in the world. A recent study indicated that the prevalence of DM in China was 11.2% (95% confidence interval 10.5% to 11.9%), especially in Han ethnicity.^1^ About 35% of patients with type 2 DM (T2DM) would eventually develop DKD, with an increased mortality,^2^ but the etiology of diabetic kidney disease is yet still unclear.

Recent studies highlighted the involvement of gut‐kidney axis in nephropathy.^3^, ^4^ Tao et al. demonstrated that gut microbiota composition was associated with the occurrence of DKD, and the individuals with DKD could be accurately distinguished from individuals with diabetes by the variables of two genera (*g_Escherichia*‐*Shigella* and *g_ Prevotella_9)*.^5^ Another study showed that fecal microbiota transplantation could reverse intestinal microbiota dysbiosis and improve renal function in rats with DKD.^6^ These suggested that gut microbiota dysbiosis may play an important role in the pathogenesis of DKD.

Besides, studies also indicated that gut microbiota and kidney were interacted *via* gut‐kidney axis, which also participated in kidney injury process. Being one of the major metabolites of microbiota‑mediated fiber fermentation process in the gut, short‐chain fatty acids (SCFAs) have attracted considerable interest. SCFAs are a subset of fatty acids that contain 6 or less carbon molecules and have shown beneficial effects on kidney.^4^, ^7^ SCFAs played a role in biological modulation by attenuating the inflammatory response and reducing mean arterial pressure, via inhibiting histone deacetylases (HDACs) and activating G protein receptor 41(GPR41), GPR43, GPR109a, and Olfr78.^8^, ^9^ However, SCFAs presented markedly varied concentrations in different diseases.^10^, ^11^ The change of fecal and serum SCFAs levels in DKD remains unclear.

In this study, all 90 participants were included from HwaMei Hospital. Fecal and serum samples were measured for SCFAs with gas chromatograph‐mass spectrometry (GC‐MS). We reported the substantial variations in the levels of fecal and serum SCFAs among normal controls, participants with diabetes, and participants with DKD. SCFA levels in participants with diabetic kidney disease were further analyzed within subgroups by renal function.

## 2 MATERIALS AND METHODS

### 2.1 Participants

There were 30 participants with DKD, 30 participants with type 2 diabetes, and 30 normal controls included in HwaMei Hospital, University of Chinese Academy of Science from January 1, 2018 to December 31, 2019. The diagnosis of T2DM was defined by the criterion issued by American Diabetes Association (ADA) in 2017.^12^ Diabetic kidney disease can be diagnosed when patients with type 2 diabetes meet any of the following situations: (1) macroalbuminuria; (2) microalbuminuria with diabetic retinopathy.^13^ All participants were on an omnivorous diet and none of the subjects reported special dietary habits. Besides, all of them underwent a medical history screening, a physical examination, and body mass index (BMI) was calculated. Lab tests were complete blood count and metabolic panel including albumin, fasting glucose, lipid profile, renal function, and urinary albumin creatinine ratio (UACR). Estimated glomerular filtration rate (eGFR) was calculated with the CKD‐EPI_Scr_ formula. Participants in the NC group from physical examination center were given tests including metabolic panel, urinalysis, stool test, HBsAg (hepatitis B surface antigen), and anti‐HCV (hepatitis C antibody). Exclusions include: receiving antibiotics, probiotics, taking laxatives, or yogurt within 2 months, gastrointestinal or systemic diseases known to affect gut bacterial composition, primary or other secondary kidney diseases, obesity, liver cirrhosis with/without complications, non‐alcoholic fatty liver disease, HBsAg, or anti‐HCV positive. The clinical parameters are shown in Table 1. The flow diagram is shown in Figure S1. The research protocols were conformed to the provisions of the Declaration of Helsinki and were approved by the Ethic Committee of Ningbo No.2 Hospital (No.2017–055–01). Informed consent for the study and the publication was obtained from each participant.

**TABLE 1:** Baseline clinical characteristics of participants

characteristics	NC(n=30)	DM(n=30)	DKD(n=30)	*P* value
Age(years)‡	51.93±8.62	59.10±8.45^a^	61.17±8.09^b^	<0.001**
Gender, male(n, %)	15(50%)	19(63.3%)	24(80%)	0.052
Duration of the disease(years)‡	‐‐	8.22±7.41	12.43±6.24	0.022*
Body mass index(BMI, Kg/m^2^)‡	23.51±2.33	24.70±5.96	25.29±3.68	0.275
Hb(g/L)^†^	142.50(133.75,155.00)	138.50(128.00,153.50)	115.50(96.00,135.75)^b^, ^c^	<0.001**
CRP(mg/L)‡	‐‐	3.15±3.40	1.89±2.30	0.160
Glucose(mmol/L)^†^	5.16(4.83,5.52)	5.79(5.06,8.34)^a^	6.11(5.15,7.42)^b^	0.001**
HbA1c(mmol /mol)‡	‐‐	70±2	59±2	0.080
HbA1c(%)‡	‐‐	8.56±1.96	7.54±1.99	0.080
TC(mmol/L)^†^	4.71(4.27,5.10)	4.35(3.34, 4.96)	4.71(3.38, 5.73)	0.199
TG(mmol/L)‡	1.24±0.75	1.87±2.21	1.96±1.60	0.181
HDL (mmol/L)‡	1.46±0.31	1.08±0.25^a^	1.12±0.37^b^	<0.001**
LDL(mmol/L)^†^	2.77(2.35,3.15)	2.71(2.16,3.31)	2.82(1.84,3.09)	0.863
Alb(g/L)‡	47.11±5.97	42.14±3.79^a^	38.43±5.68^b^	<0.001**
BUN(mmol/L)^†^	4.58(4.23,5.70)	5.29(4.61,7.09)	8.77(5.53,16.85)^b^, ^c^	<0.001**
UA(μmol/L)‡	315.44±79.69	331.32±76.69	378.31±125.96^b^	0.039*
Creatinine(μmol/L)^†^	61.30(52.50,73.05)	54.55(49.25,67.85)	107.10(63.68,266.63)^b^, ^c^	<0.001**
eGFR(ml/min/1.73m^2^)^†^	102.67(98.56,110.81)	102.29(96.72,110.41)	64.60(18.03,95.99)^b^, ^c^	<0.001**
UACR(mg/g)^†^	‐‐	5.30(2.30, 22.00)	789.55(354.43, 2097.70)^c^	<0.001**
Metformin(n, %)	‐‐	17(56.7%)	16(53.3%)	0.795

Abbreviations: NC, normal controls; DM, diabetic mellitus; DKD, diabetic kidney disease; Hb, hemoglobin; CRP, C‐reactive protein; HbA1c, hemoglobin A1c; TC, total cholesterol; TG, triglyceride; HDL, high‐density lipoprotein; LDL, low‐density lipoprotein; Alb, albumin; BUN, blood urea nitrogen; UA, uric acid; eGFR, estimated glomerular filtration rate; UACR, urine albumin creatinine ratio.

^‡^ Data are expressed as mean±standard error.

^a^ *P*<0.05 DM compared to NC.

^b^ *P*<0.05 DKD compared to NC.

^c^ *P*<0.05 DKD compared to DM.

^†^ Data are expressed as median (p25th‐p75th).

* *p*<0.05

** *p*<0.01.

### 2.2 Fecal and serum sample collection

Fresh fecal samples were collected and a portion of 200mg was utilized for each test. Blood samples were collected in the fasting status and serum was obtained by centrifugation at 3,500rpm for 5min at 4℃. These samples were then stored at −80°C until usage. One fecal sample and one serum sample in DKD group were later found not usable and were excluded in the study. 30 serum samples in NC group were not collected from the physical examination center. Hence, 30 fecal samples in NC group, 30 fecal and serum samples in the diabetes group, and 29 fecal and serum samples in the group with DKD were used for data determination.

### 2.3 Fecal and serum sample processing

Each fecal sample of 200mg was mixed with 0.8mL of ultrapure water, crushed with a tissue grinder, and then centrifuged at 12,000 rpm for 20 min at 4℃. Each 0.4ml supernatant was mixed with 0.1mL of 50% sulfuric acid (ultrapure water diluted), 0.5ml of ether (containing 50μg/mL of internal standard dimethyl valeric acid) for 1 min, centrifuged at 12,000 rpm for 20 min at 4℃, and then stood for 30 min at 4℃. The supernatant ether layer was filtered through anhydrous sodium sulfate for GC–MS analysis.

Each serum sample (100μL) was mixed with 50μL of 50% sulfuric acid (ultrapure water diluted), 200μL of ether (containing standard dimethyl valeric acid) for 1 min, centrifuged at 12,000 rpm for 20 min at 4℃, and then stood for 30 min at 4℃. The supernatant ether layer was filtered through anhydrous sodium sulfate and the solution later transferred to a glass vial for GC–MS analysis.

### 2.4 Determination of SCFAs using gas chromatograph‐mass spectrometry (GC‐MS)

The analysis was performed using the GC‐MS 7890A‐5975C (Agilent Technology, USA). A FFAP capillary column (30m×0.25mm×0.25μm) was used for chromatographic separation, and helium (1 mL/min) was used as the carrier gas. The stepwise chromatographic thermal conditions were as follows: 100°C for 1 min, 5°C/min to 160°C, 40°C/min to 240°C, maintaining for 10 min. The mass spectrometer was set to scan mode at m/z 100–300 and selected ion monitoring mode at m/z 60 for acetate, butyrate, iso‐valerate, valerate, and caproate, maintaining for 4.72min、7.34min、8.90min、8.03min, and 11.26min respectively, as well as m/z 73 for propionate and iso‐butyrate for 5.90min and 6.31min separately.

### 2.5 Statistical analysis

All statistical analyses were performed with SPSS Statistics 19.0 (SPSS Inc., Chicago, IL, USA) and GraphPad Prism 7.0. The results were expressed as means with standard deviation (SD) for normally distributed continuous variables, median values (interquartile ranges) for non‐normally distributed continuous variables, and frequencies and percentages for categorical variables. ANOVA or Student's t‐test for independent samples was used for normally distributed continuous variables. Comparisons of non‐normally distributed continuous variables were performed using the Mann‐Whitney U‐test or Kruskal‐Wallis test. For categorical variables, the chi‐square test was used. Correlation difference between variables was analyzed by Spearman's R coefficient using psych package 1.9.12, and visualized by heatmap in corrplot package 0.84. The association between fecal or serum level with the clinic index was examined via binary logistic regression analysis, based on median level of fecal or serum SCFAs. Covariates with *p*<0.1 in the univariate regression analysis were chosen for multivariate regression analysis. A *P* value<0.05 was considered statistically significant.

## 3 RESULTS

### 3.1 Baseline characteristics among the three groups

Baseline clinical and biochemical characteristics of all participants in NC group, DM group, and DKD group are shown in Table 1. Among the three groups, the levels of total cholesterol, triglyceride, and low‐density lipoprotein were similar without statistical significance. The percentage of participants using metformin, sodium‐dependent glucose transporters 2 (SGLT‐2) had no difference between DM group and DKD group. Besides, no participants took the tablets of the phosphorus chelators that may influence the experimental results.

### 3.2 Comparisons of fecal and serum SCFAs among the three groups

**FIGURE 1:**
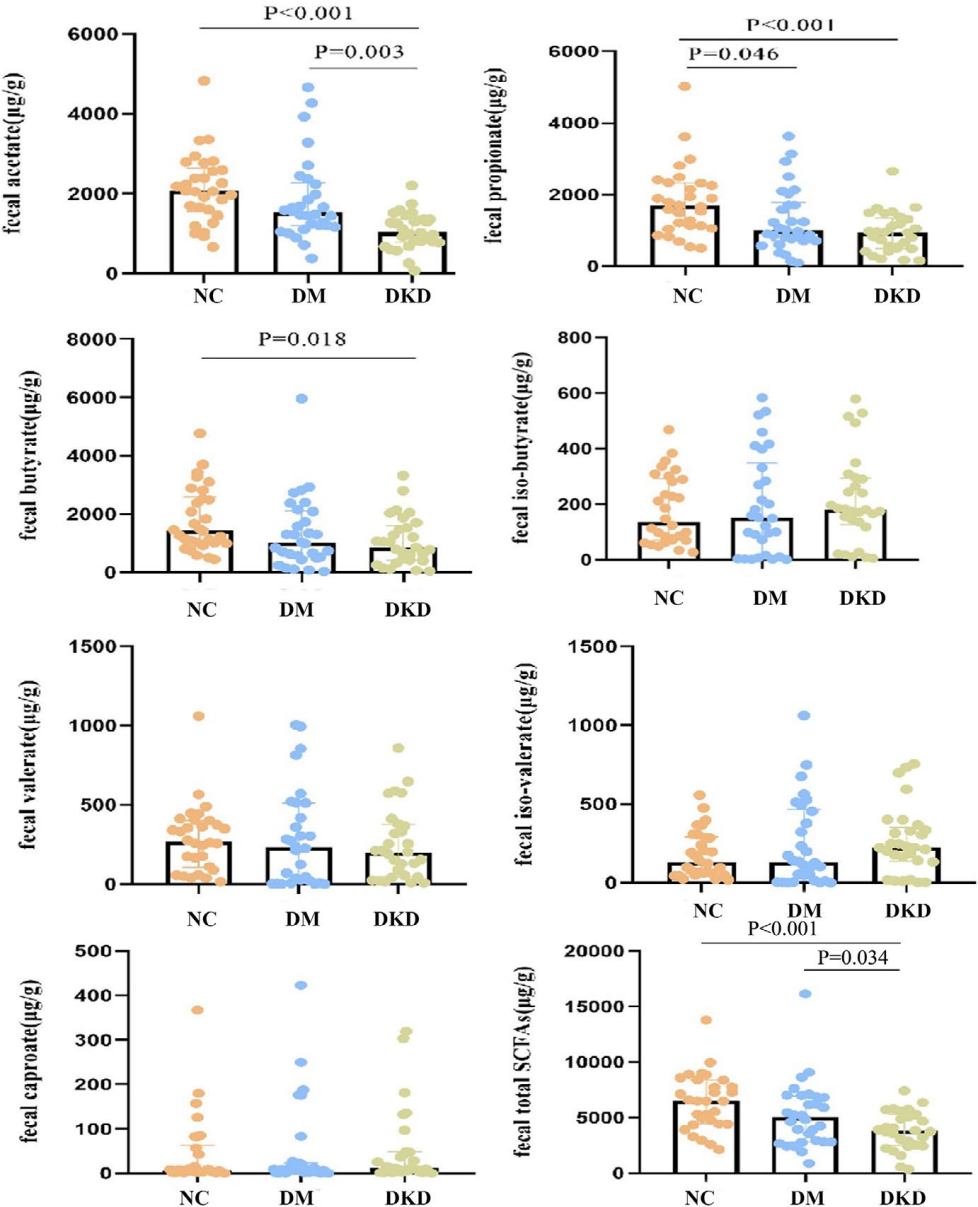
The concentrations of fecal SCFAs in NC, DM and DKD groups

The acetate, propionate, butyrate, iso‐butyrate, valerate, iso‐valerate, and caproate in stool sample were identified (Figure 1). Notably, the content of acetate in the stool was markedly lower in the group with DKD versus DM group (*p*=0.003) and NC group (*p*<0.001). Lower propionate and butyrate levels in DKD group were observed compared with NC group (*p*<0.05). Correspondingly, fecal total SCFAs presented in the same trend, being lowest in DKD group, 3843.01 (2491.81–5290.88) μg/g, while highest in NC group, reaching 6482.68 (4438.91–8379.59) μg/g (*p*<0.001). However, the median levels of iso‐butyrate, valerate, iso‐valerate, and caproate were equivalent among the three groups (*p*>0.05).

**FIGURE 2:**
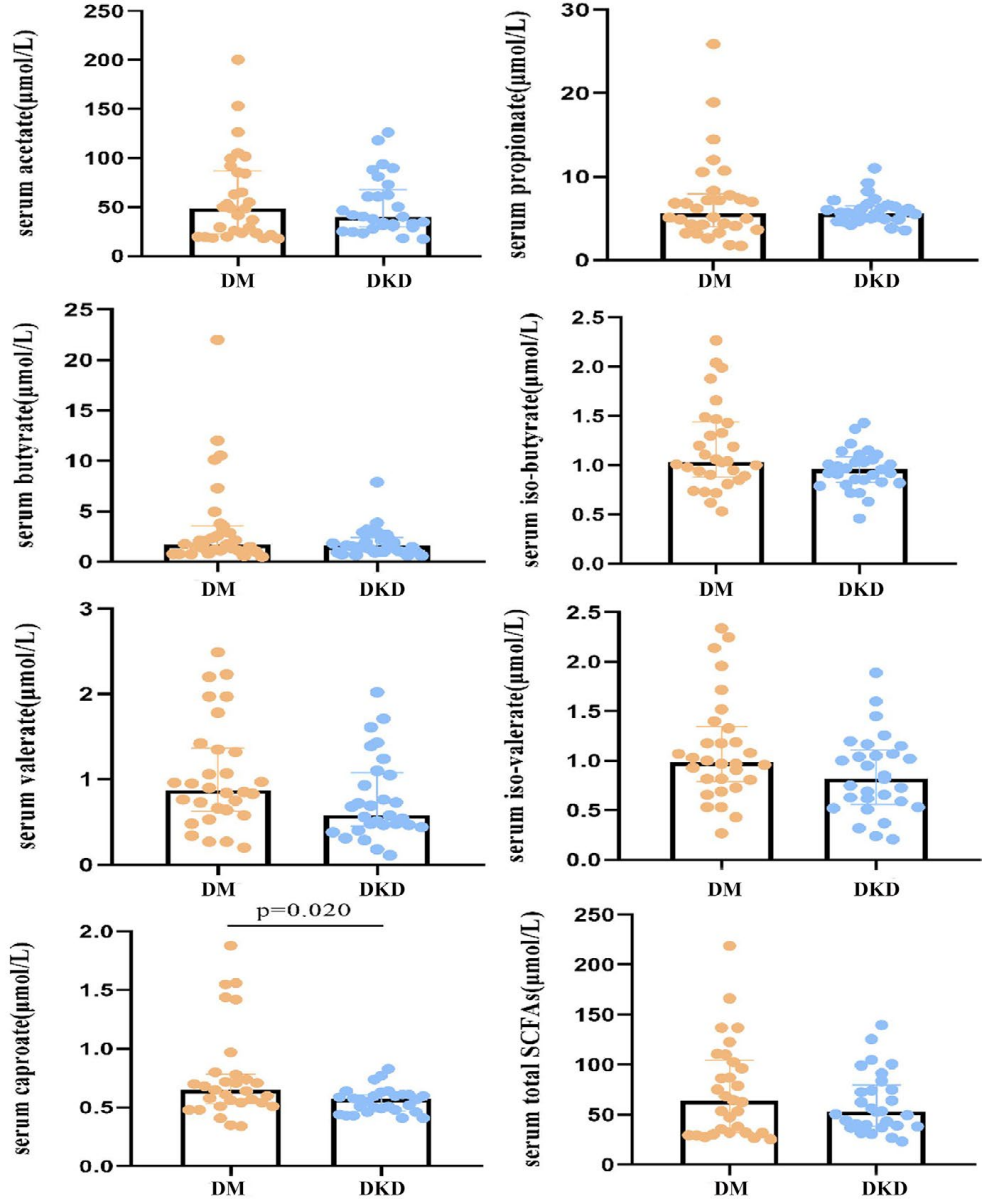
The concentration of serum SCFAs in DM, and DKD groups

Meanwhile, serum SCFAs were also measured in DM and DKD groups (Figure 2). We observed a significant difference in serum caproate in DM group [0.65(0.53–0.79) μmol/L] versus DKD group [0.57(0.47–0.61) μmol/L] (*p*<0.05). In addition, the differences of the concentrations of serum iso‐butyrate, valerate, and iso‐valerate between DKD group and DM group were approaching statistical significance, which were lower in DKD group (*p*=0.081, *p*=0.050, *p*=0.070, respectively). Apart from this, other SCFAs between DM group and DKD group showed no difference. Unexpectedly, there was no correlation between serum SCFAs and corresponding fecal SCFAs (raw *p*>0.05).

### 3.3 The correlations between SCFAs and the biochemical indicators

**FIGURE 3:**
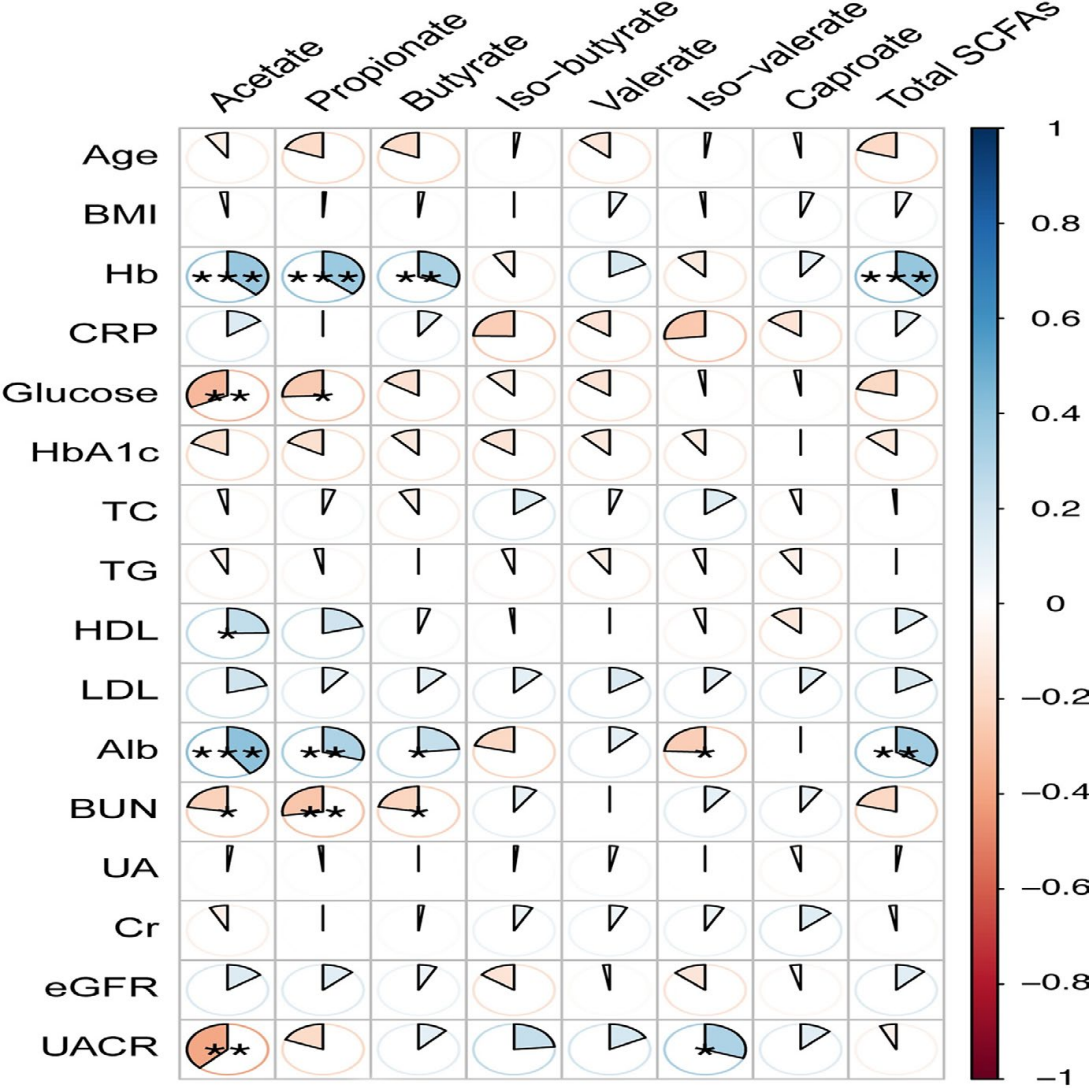
The correlations between fecal SCFAs and the biochemical indicators ***p<0.001, **p<0.01, *p<0.05. Abbreviation: Hb, hemoglobin; CRP, C‐reactive protein; HbA1c, hemoglobin A1C; TC, total cholestrol; TG, triglyceride; HDL, high density lipoprotein; LDL, low density lipoprotein; Alb, albumin; BUN, blood urea nitrogen; UA, uric acid; eGFR, estimated glomerular filteration rate; UACR, urine albumin creatinine ratio. Blood urea nitrogen realted with fecal acetate, propionate and butyrate negatively(r=−0.22, *p*=0.03; r=−0.27, *p*<0.01; r=−0.21, *p*=0.03, respectively), UACR related with fecal acetate negatively (r=−0.38, *p*<0.01)), hemoglobin and serum albumin level related with fecal acetate, propionate and butyrate positively (*p*<0.05), bloos glucose realted with fecal acetate and propionate negatively (r=−0.32; *p*<0.01; r=−0.25; *p*=0.01, respectively)

Correlations between the fecal SCFAs and clinical indicators were estimated by Spearman's correlation analysis (Figure 3). As expected, an inverse relationship was observed between blood urea nitrogen and fecal acetate, propionate, and butyrate levels (r=−0.22, *p*=0.03; r=−0.27, *p*<0.01; r=−0.21, *p*=0.03, respectively). Meanwhile, UACR was negatively related to fecal acetate (r=−0.38, *p*<0.01). Interestingly, hemoglobin and serum albumin levels showed a positive relationship with fecal acetate, propionate, and butyrate (*p*<0.05). Blood glucose was negatively related to fecal acetate and propionate (r=−0.32, *p*<0.01; r=−0.25, *p*=0.01, respectively).

**FIGURE 4:**
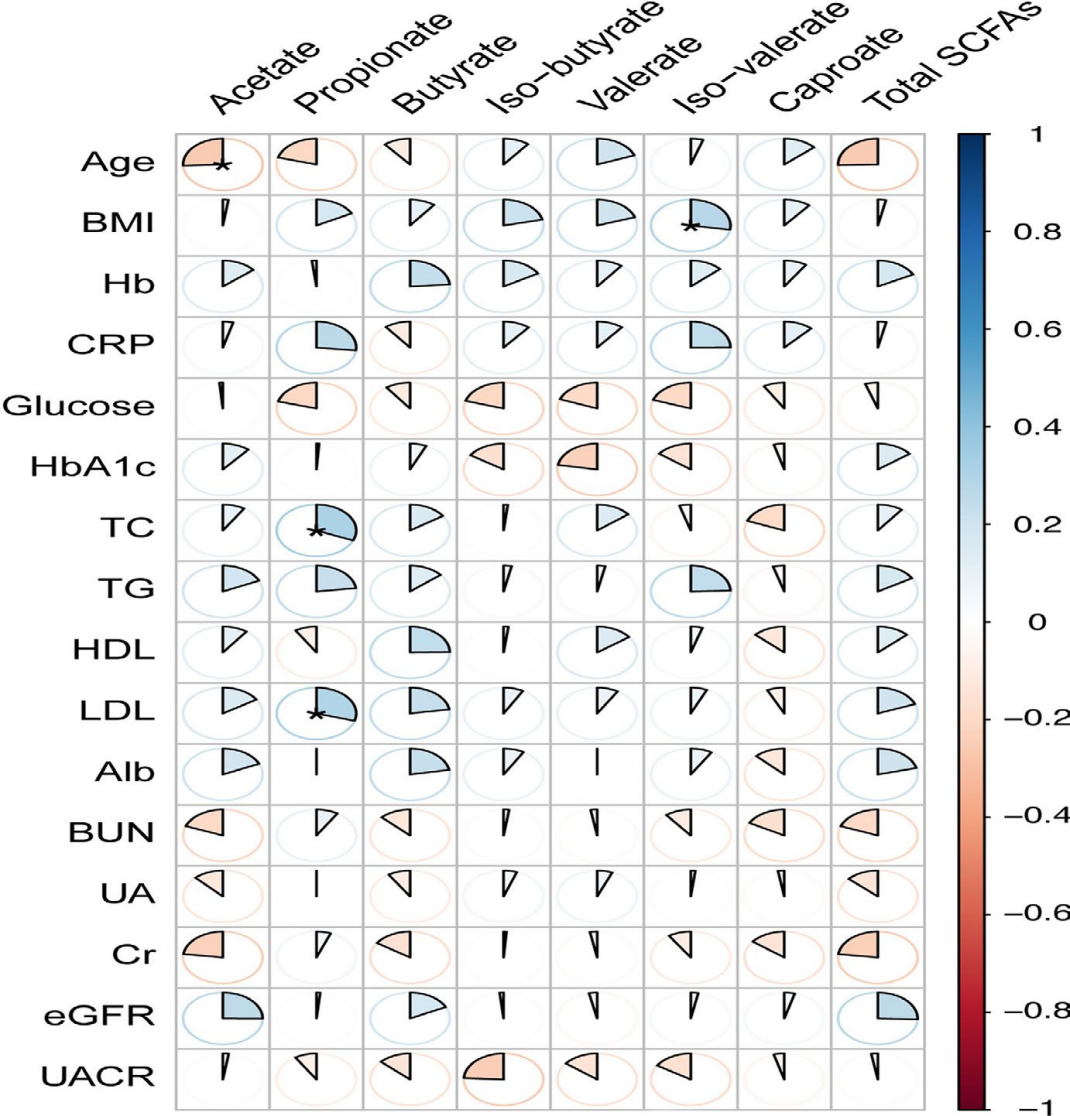
The correlations between fecal SCFAs and the biochemical indicators ***p<0.001, **p<0.01, *p<0.05. Abbreviation: Hb, hemoglobin; CRP, C‐reactive protein; HbA1c, hemoglobin A1C; TC, total cholestrol; TG, triglyceride; HDL, high density lipoprotein; LDL, low density lipoprotein; Alb, albumin; BUN, blood urea nitrogen; UA, uric acid; eGFR, estimated glomerular filteration rate; UACR, urine albumin creatinine ratio. Age related with acetate negatively(r=−0.25,*p*=0.04), total choleatrol, low density lipoprotein related with propionate positively (r=0.31, *p*=0.03; r=0.29, *p*=0.02)

We further investigated the correlations between serum SCFAs and biochemical indicators (Figure 4). Unexpectedly, no statistical correlations were found between renal function markers and serum SCFAs, except for a negative correlation between age and acetate level (r=−0.25, *p*=0.04), positive correlations between total cholesterol, low‐density lipoprotein, and propionate (r=0.31, *p*=0.03; r=0.29, *p*=0.02).

In the univariate regression analysis, fecal acetate and serum acetate were both correlated with eGFR [OR=1.013, 95%CI (0.999, 1.028), *p*=0.072; OR=1.017, 95%CI (1.002, 1.034), *p*=0.032] (Tables 2 and 3). However, in multivariate analysis, acetate in stool (Table 2) or serum (Table 3) showed no correlation with eGFR (*p*>0.05). Total SCFAs correlated with eGFR in subjects with statistical significance [OR=1.019, 95%CI (1.002, 1.035), *p*=0.024] unadjusted while the correlation became borderline significant [OR=1.024, 95%CI (0.999, 1.050), *p*=0.063] (Table 4) when adjusted for Hb and LDL. Interestingly, fecal acetate, serum acetate, and total SCFAs each related with Hb in subjects with statistical significance [OR=1.032, 95%CI (1.009, 1.056), *p*=0.007; OR=1.026, 95%CI (1.000, 1.052), *p* =0.049; OR=1.027, 95%CI (1.002, 1.054), *p*=0.038] (Tables 2, 3, 4).

**TABLE 2:** Univariate and multivariate associates of fecal acetate in participants

Variables	Fecal acetate					
	Univariable			Multivariate		
	OR	95%CI	*P* value	OR	95%CI	*P* value
**Age (year)**	0.966	(0.922,1.013)	0.153			
**Gender**	0.966	(0.406,2.295)	0.937			
**Body mass index**	0.915	(0.801,1.046)	0.192			
**Hb**	1.032	(1.009,1.056)	0.007**	1.041	(1.001,1.083)	0.046*
**CRP**	1.182	(0.953,1.466)	0.127			
**Glucose**	0.710	(0.531,0.951)	0.022*	0.705	(0.518,0.959)	0.026*
**HbA1c**	0.985	(0.956,1.014)	0.309			
**TC**	0.895	(0.591,1.355)	0.601			
**TG**	0.740	(0.491,1.118)	0.153			
**HDL**	6.016	(1.498,24.163)	0.011*	6.180	(1.288,29.642)	0.023*
**LDL**	1.185	(0.677,2.076)	0.552			
**Alb**	1.141	(1.036,1.257)	0.007**	1.027	(0.918,1.148)	0.643
**BUN**	0.947	(0.873,1.028)	0.193			
**UA**	1.001	(0.996,1.005)	0.771			
**Creatinine**	0.998	(0.995,1.002)	0.366			
**eGFR**	1.013	(0.999,1.028)	0.072	1.002	(0.979,1.025)	0.875
**UACR**	1.000	(0.999,1.000)	0.154			

Abbreviations: Hb, hemoglobin; CRP, C‐reactive protein; HbA1c, hemoglobin A1c;TC, total cholesterol; TG, triglyceride; HDL, high‐density lipoprotein; LDL, low‐density lipoprotein; Alb, albumin; BUN, blood urea nitrogen; UA, uric acid; eGFR, estimated glomerular filtration rate; UACR, urine albumin creatinine ratio.

* *p*<0.05

** *p*<0.01.

**TABLE 3:** Univariate and multivariate associates of serum acetate in participants

Variables	Serum acetate					
	Univariable			Multivariate		
	OR	95%CI	*P* value	OR	95%CI	*P* value
**Age (year)**	0.949	(0.888,1.014)	0.120			
**Gender**	1.571	(0.503,4.914)	0.437			
**Body mass index**	1.085	(0.942,1.249)	0.258			
**Hb**	1.026	(1.000,1.052)	0.049*	1.012	(0.978, 1.047)	0.506
**CRP**	1.128	(0.896,1.421)	0.306			
**Glucose**	0.848	(0.696,1.032)	0.100			
**HbA1c**	0.987	(0.742,1.311)	0.926			
**TC**	1.112	(0.717,1.725)	0.636			
**TG**	1.038	(0.790,1.363)	0.790			
**HDL**	0.702	(0.131,3.767)	0.680			
**LDL**	1.520	(0.808,2.858)	0.194			
**Alb**	1.064	(0.959,1.180)	0.244			
**BUN**	0.898	(0.809,0.996)	0.042*			
**UA**	0.998	(0.993,1.003)	0.401			
**Creatinine**	0.995	(0.991,1.000)	0.070			
**eGFR**	1.017	(1.002,1.034)	0.032*	1.013	(0.991, 1.034)	0.246
**UACR**	1.000	(0.999,1.000)	0.317			

Abbreviations: Hb, hemoglobin; CRP, C‐reactive protein; HbA1c, hemoglobin A1c; TC, total cholesterol; TG, triglyceride; HDL, high‐density lipoprotein; LDL, low‐density lipoprotein; Alb, albumin; BUN, blood urea nitrogen; UA, uric acid; eGFR, estimated glomerular filtration rate; UACR, urine albumin creatinine ratio.

* *p*<0.05

** *p*<0.01.

**TABLE 4:** Univariate and multivariate associates of serum total SCFAs in participants

Variables	Serum total SCFAs					
	Univariable			Multivariate		
	OR	95%CI	*P* value	OR	95%CI	*P* value
**Age (year)**	0.948	(0.887,1.013)	0.113			
**Gender**	1.571	(0.503,4.914)	0.437			
**Body mass index**	1.060	(0.934,1.202)	0.365			
**Hb**	1.027	(1.002,1.054)	0.038*	0.996	(0.958,1.034)	0.817
**CRP**	1.118	(0.891,1.403)	0.336			
**Glucose**	0.861	(0.713,1.039)	0.119			
**HbA1c**	0.997	(0.750,1.326)	0.984			
**TC**	1.245	(0.795,1.949)	0.338			
**TG**	1.033	(0.787,1.356)	0.812			
**HDL**	0.784	(0.147,4.170)	0.775			
**LDL**	1.895	(0.970,3.702)	0.061	2.381	(1.068,5.305)	0.034
**Alb**	1.090	(0.979,1.214)	0.115			
**BUN**	0.885	(0.792,0.989)	0.031*			
**UA**	0.998	(0.993,1.003)	0.479			
**Creatinine**	0.995	(0.990,1.000)	0.066			
**eGFR**	1.019	(1.002,1.035)	0.024*	1.024	(0.999,1.050)	0.063
**UACR**	1.000	(0.999,1.000)	0.355			

Abbreviations: Hb, hemoglobin; CRP, C‐reactive protein; HbA1c, hemoglobin A1c; TC, total cholesterol; TG, triglyceride; HDL, high‐density lipoprotein; LDL, low‐density lipoprotein; Alb, albumin; BUN, blood urea nitrogen; UA, uric acid; eGFR, estimated glomerular filtration rate; UACR, urine albumin creatinine ratio.

* *p*<0.05

** *p*<0.01.

### 3.4 The subgroup analysis of fecal and serum SCFAs in DKD

To study the fecal and serum SCFAs in patients with various renal function, we categorized the DKD patients into two subgroups according to the eGFR level, the low GFR subgroup (eGFR<60ml/min, n=14), and the high GFR subgroup (eGFR≥60ml/min, n=16). The baseline data of the two subgroups were shown in Table S1. Age, gender, and BMI between the two groups were matched with no statistical difference (*p*>0.05). UACR, serum creatinine, and blood urea nitrogen were higher (*p*<0.05) in the low GFR subgroup compared with the high GFR subgroup with statistically significant difference.

There were no differences in fecal SCFAs between the two subgroups (*p*>0.05). As shown in Table S2, serum acetate and total SCFAs were lower and with borderline significant in the low GFR subgroup versus the high GFR subgroup (*p*=0.055, *p*=0.050, respectively). However, other SCFAs had no difference between these two subgroups (*p*>0.05).

## 4 DISCUSSION

It is the first study to investigate fecal and serum SCFAs simultaneously in individuals with DKD. In this study, fecal acetate, propionate, butyrate, and total SCFAs were markedly lower in the DKD group. Serum acetate and total SCFAs were also found lower in the low GFR subgroup. Furthermore, fecal and serum acetate seem to be respectively correlated with eGFR in DKD patients. Besides, serum total SCFAs seem to be an independent factor for renal function.

SCFAs are end products of bacterial carbohydrate fermentation, and function as an important energy source and signaling molecules.^14^ The concentration of SCFAs varies among different diseases. In DKD mice, there was a significant decrease in propionic acid and butyric acid contents in DKD progression.^15^ The study conducted by Wang et al showed that fecal SCFAs declined in CKD patients, and negatively correlated with the renal function.^16^ It was consistent with our study that SCFAs, mainly acetate, propionate, and butyrate levels were evidently lower in DKD patients compared to DM and NC groups.

The gut microbiota, yielding SCFAs as the major products, was also believed to involve with DKD. Studies have clearly outlined the changes in microbiota in DKD patients,^5^, ^17^ that the richness of gut microbiota and the variation of bacteria population were found different in DKD compared to DM^5^ and SCFAs‐producing bacteria *Prevotella* declining in DKD patients.^5^ We speculated that this reduction of SCFAs‐producing bacteria was accompanied by the decrease of yielding SCFAs. Maybe this was the result of the lowest fecal SCFAs levels in DKD.

Despite the finding of fecal SCFAs changes, there has not been a defined study on the subsequent serum SCFAs in DKD patients. Our study revealed that the serum acetate was lower in the low GFR subgroup than in the high GFR subgroup with a borderline significant difference. This change is postulated to be caused by changes in medication, gastrointestinal microecology, and host physiology and pathology. However, we noticed that the main types of SCFAs, including acetate, propionate, butyrate, and valerate did not change significantly in DKD group versus DM group, which was unexpected given recent literature identifying a significant decline in SCFAs‐producing bacteria with advancing kidney disease.^17^ Wang et al demonstrated that serum acetate and butyrate level was significantly lower in CKD 5 patients than in CKD 1–4 patients.^16^ Jadoon et al found a significant graded decrease in the concentration of acetate, but the plasma valerate concentration increased in patients with advancing kidney disease than in mild CKD patients.^18^ Paradoxically, in streptozotocin (STZ)‐induced DKD rats, serum acetate levels were markedly elevated compared with controls.^6^ The conclusions indicated by our study vary from the above studies, assuming that being associated with the small sample size and the few participants with CKD 5, as well as the low peripheral concentration of SCFAs, which may mitigate the changes.^16^ Furthermore, the discrepancies of SCFAs change were possibly due to different etiology of CKD, various severities of the disease, and different animal models.^6^ Meanwhile, intestinal microecology is known to be complex and each type of bacteria plays a role when the ecology changes. Therefore, it is significant to investigate the types and concentrations of SCFAs in a larger group of DKD patients. Notably, we identified a significant decline of the level of serum caproate in DKD patients than in DM patients in our study. It in line with the study that serum caproate concentration decreased in CKD 3 patients compared to non‐CKD participants conducted by Wu et al.^11^

SCFAs diffuse through the intestinal mucosa and enter the bloodstream via the portal vein.^19^, ^20^ Samuel et al found that the intestinal absorption of SCFA seems to be influenced by the G‐protein‐coupled receptor (GPCR), which is broadly distributed in mammalian organisms.^21^ However, serum SCFAs were not in parallel with fecal SCFAs changes in DM and DKD patients in our study. It is assumed that SCFAs measured in circulation may not be utilized in fecal SCFAs excretion, therefore fecal SCFAs may be more accurate in revealing SCFAs absorption or production.^22^ Several *in vitro* and *in vivo* studies have confirmed significant disruption of the colonic, ileal, jejunal, and gastric epithelial tight junction in different models of CKD in rats and in cultured human colonocytes exposed to uremic human plasma.^23^, ^24^ Meanwhile, several observations have provided indirect evidence of increased intestinal permeability in CKD patients and animals.^25^, ^26^ A human study showed that the participants with lower fecal acetate tended to have higher acetate absorption.^22^ However, the transit time of SCFAs in the large intestine does not indicate specific phases of a certain disease. Also, the level of serum SCFAs is influenced by diet manipulations. Herein, we agree that serum SCFAs are effected by many factors and it is necessary to assess both fecal and circulating SCFAs in certain diseases to achieve a better understanding of the microbiota change.

Gut microbiota participates in the progression of metabolic diseases via its metabolites. Several studies have demonstrated that SCFAs play a protective role in kidney disease. Yang et al revealed that dietary fiber supplement significantly reversed kidney injuries in CKD mice due to increased SCFAs production from microbial fermentation.^27^ Andrade‐Oliveira et al demonstrated that intraperitoneal injection with SCFAs improved acute kidney injury (AKI) by decreasing inflammatory cytokines and chemokines locally and systemically via suppressing NF‐κB signaling pathway.^4^ Huang et al found that exogenous SCFAs, especially butyrate, improved hyperglycemia and insulin resistance; prevented the formation of proteinuria and an increase in serum creatinine, urea nitrogen, and cystatin C; inhibited mesangial matrix accumulation and renal fibrosis.^28^ In the recent studies, SCFAs played an important effect on multiple aspects of renal physiology, inhibiting inflammation, immunity, and fibrosis, decreasing blood pressure, and adjusting energy metabolism.^29^

Protective effects of SCFAs on DKD have also been reported, via activation of GPCRs and the inhibition of HDAC activity. Administration of sodium butyrate (NaBu), the major members of SCFAs, ameliorates mesangial matrix expansion, fibrosis, and inflammation in the kidneys of STZ‑induced diabetic rats.^30^, ^31^ *In vitro* study, NaBu acted as an antioxidant in HG‑induced NRK‑52E cells and suppressed HG‑induced apoptosis of NRK‑52E cells through inhibiting HDAC2.^32^ *In vivo* study, dietary fiber protected against DKD through modulation of the gut microbiota, enriched SCFAs‐producing bacteria, and increased SCFA production, so that it reduced expression of genes encoding inflammatory cytokines, chemokines, and fibrosis‐promoting proteins in diabetic kidneys via GPR43 and GPR109A.^33^Recent studies found GPR41 and GPR43 protein expressed in the distal renal tubules and collecting tubules, and found SCFAs lowered TNF‐α induced MCP‐1 expression by reducing phosphorylation of p38 and JNK in a GPR41/43‐dependent manner in human renal cortical epithelial cells (HRCEs).^34^ Besides, Huang et al demonstrated that SCFAs, especially butyrate, partially improved T2D‐induced kidney injury via GPR43‐mediated inhibition of oxidative stress and NF‐κB signaling.^28^ Iso‐butyrate, valerate and iso‐valerate, have not been studied as extensively as other SCFAs, and details of the physiological effects are sparse. Previous work has identified these as ligands for GPCR,^35^ which influence a variety of metabolic, immune, and vascular processes.^36^

In this study, we did not use nutrition diaries, but all participants were interviewed for dietary habits and were explicitly asked for special dietary habits. Since all participants reported a Chinese omnivorous diet without any special dietary habits, dietary habits were unlikely to be a major confounder in the investigated subjects. However, there are some limitations in our cross‐section study, consequently we could not demonstrate the causal relationship between fecal, serum SCFAs, and the presence of DKD. This monocentric study included a small number of patients in China, prudence needs to be taken when trying to extrapolate our data to other populations. Besides, the composition and construction of gut microbiota in participants were not analyzed, therefore the relationship between fecal and serum SCFAs and gut microbiota was not identified.

In conclusion, this study provides evidence for quantitative reduction of gut microbial products‐SCFAs (fecal acetate, propionate, and butyrate in particular) in DKD patients, demonstrating the association of SCFAs with renal function in DKD.

## CONFLICT OF INTERESTS

The authors declare that they have no conflicts of interest.

## AUTHOR CONTRIBUTIONS

Study conception and design: KC, CZ, QL; Administrative support: KC, QL. Patient education and instruction: KC, CZ, LW, JL, WG, QZ, JZ; Collection and assembly of data: KC, CZ, ZD, LC, LW, JL, WG, QZ, JZ, CX, HL; Data analysis and interpretation: KC, CZ, QL; K and CZ took the lead in writing/wrote the manuscript with input from all authors. Manuscript writing: All authors. Final approval of manuscript: All authors.

## Supporting information

Figure S1

Figure S2

Table S1‐S2

## Data Availability

All data generated and/or analyzed during this study are available from the corresponding authors upon reasonable request.
