# Supplementary figures and images for: The change of gut microbiota‐derived short‐chain fatty acids in diabetic kidney disease
Source: J Clin Lab Anal. 2021 Oct 24;35(12):e24062. doi: 10.1002/jcla.24062 (PMC8649351; doi:10.1002/jcla.24062)

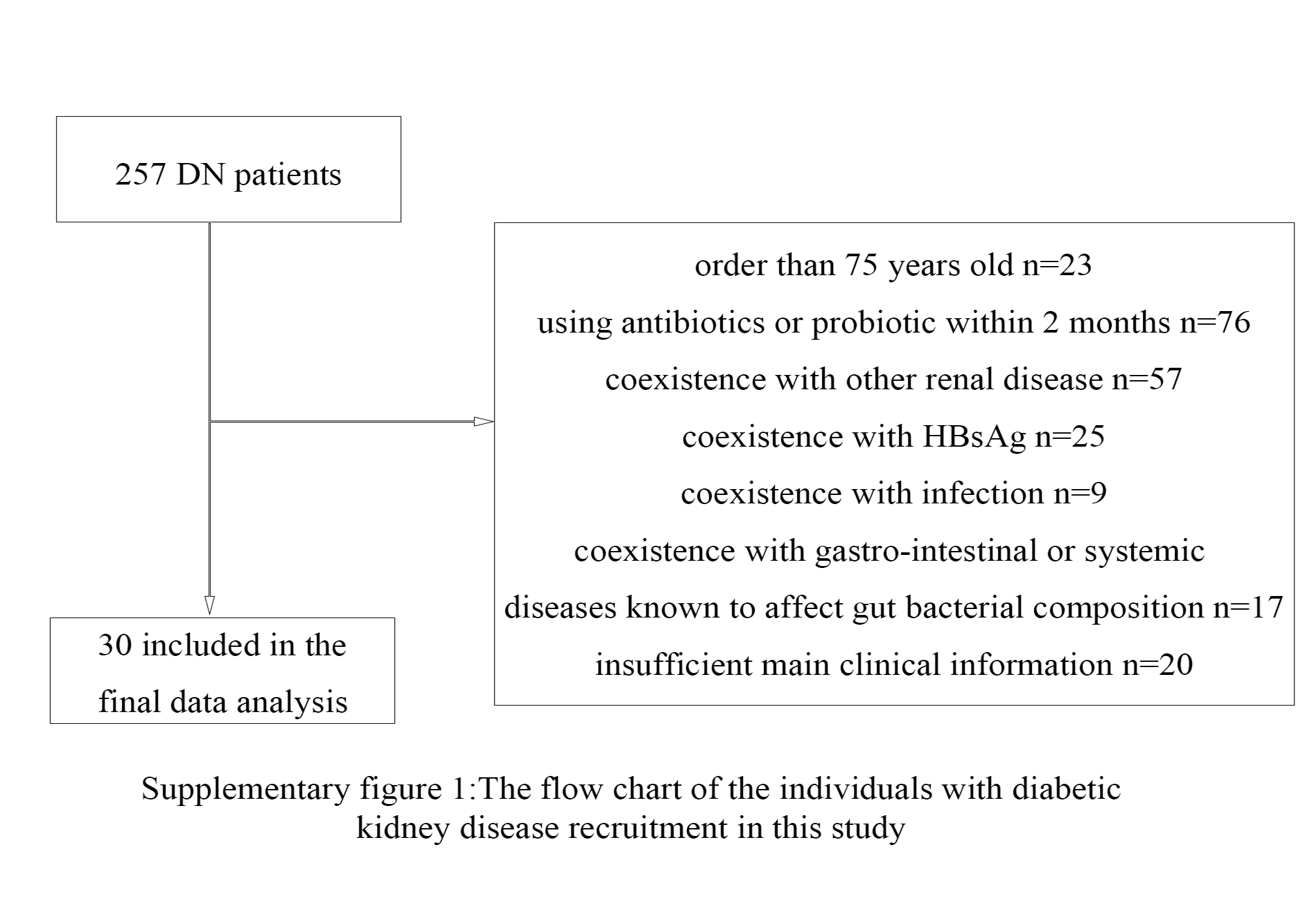

Supplement: Supplementary file 1 — Figure S1 [file JCLA-35-e24062-s002.png]

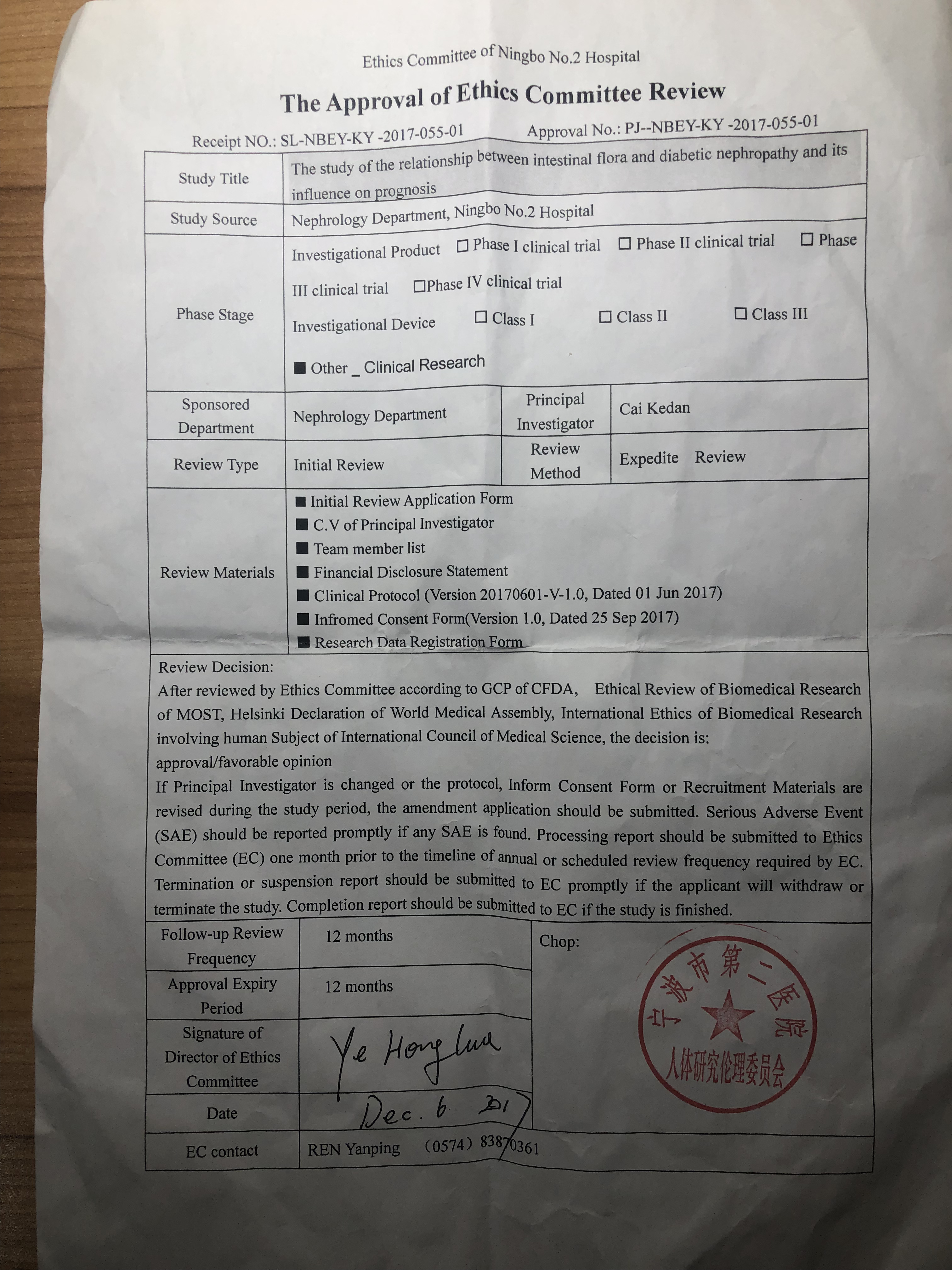

Supplement: Supplementary file 2 — Figure S2 [file JCLA-35-e24062-s003.png]
