# Supplementary material for: The change of gut microbiota‐derived short‐chain fatty acids in diabetic kidney disease
Source: J Clin Lab Anal. 2021 Oct 24;35(12):e24062. doi: 10.1002/jcla.24062 (PMC8649351; doi:10.1002/jcla.24062)
Supplement: Supplementary file 3 — Table S1‐S2 [file JCLA-35-e24062-s001.docx]

Supplementary Table 1:Baseline clinical characteristics in the group with diabetic nephropathy

| characteristics | eGFR≥60(n=16) | eGFR<60(n=14) | *P* value |
| --- | --- | --- | --- |
| Age(years)‡ | 60.56±8.73 | 61.86±7.56 | 0.670 |
| Gender,male(n, %) | 12(75.0%) | 12(85.7%) | 0.464 |
| Duration of the disease(years)‡ | 10.56±6.44 | 14.57±5.46 | 0.079 |
| Body mass index(BMI, Kg/m^2^)‡ | 25.89±3.60 | 24.60±3.78 | 0.350 |
| Hb(g/L)‡ | 129.50±17.74 | 101.29±18.21 | <0.001** |
| CRP(mg/L)‡ | 2.13±3.00 | 1.61±1.10 | 0.563 |
| Glucose(mmol/L)‡ | 7.06±3.51 | 6.65±2.97 | 0.739 |
| HbA1c(mmol /mol)‡ | 62±0 | 56±3 | 0.481 |
| HbA1c(%)‡ | 7.81±2.11 | 7.26±1.90 | 0.481 |
| TC(mmol/L)‡ | 4.55±1.76 | 4.93±1.10 | 0.512 |
| TG(mmol/L)‡ | 2.19±2.07 | 1.69±0.69 | 0.409 |
| HDL (mmol/L)‡ | 1.13±0.26 | 1.12±0.48 | 0.934 |
| LDL(mmol/L)‡ | 2.54±1.07 | 2.88±0.90 | 0.366 |
| Alb(g/L)‡ | 39.69±5.55 | 36.87±5.66 | 0.188 |
| BUN(mmol/L)‡ | 6.25±2.91 | 17.84±7.77 | <0.001** |
| UA(μmol/L)‡ | 350.26±129.32 | 412.82±117.38 | 0.188 |
| Creatinine(μmol/L)‡ | 70.94±26.35 | 320.59±185.87 | <0.001** |
| eGFR(ml/min/1.73m^2^)‡ | 92.81±20.93 | 25.37±17.54 | <0.001** |
| UACR(mg/g)‡ | 867.07±1095.82 | 2258.47±1904.55 | 0.027* |
| Metformin(n, %) | 12(75.0%) | 4(28.6%) | 0.011* |

Abbreviation: Hb, hemoglobin; CRP, C-reactive protein; HbA1c, hemoglobin A1c;TC, total cholesterol; TG, triglyceride; HDL, high-density lipoprotein; LDL, low-density lipoprotein; Alb, albumin; BUN, blood urea nitrogen; UA, uric acid; eGFR, estimated glomerular filtration rate;UACR, urine albumin creatinine ratio.

**P*<0.05, ***P*<0.01

‡ Data are expressed as mean±standard error.

Supplementary Table 2: The concentration of serum SCFAs in the group with diabetic nephropathy

| SCFA types | eGFR≥60(n=16) | eGFR<60(n=14) | *P* value |
| --- | --- | --- | --- |
| acetate(μmol/L)**†** | 60.71(31.43, 89.92) | 33.79(27.04, 47.28) | 0.055 |
| propionate(μmol/L)**†** | 5.22(4.65,6.41) | 5.72(5.35,6.66) | 0.326 |
| butyrate(μmol/L)**†** | 1.57(1.00,2.94) | 1.26(0.93,1.90) | 0.138 |
| Iso-butyrate(μmol/L)‡ | 0.93±0.23 | 0.98±0.18 | 0.576 |
| valerate(μmol/L)**†** | 0.56(0.44,0.93) | 0.63(0.45,1.48) | 0.419 |
| Iso-valerate(μmol/L)‡ | 0.89±0.38 | 0.82±0.44 | 0.649 |
| caproate(μmol/L)‡ | 0.54±0.07 | 0.58±0.13 | 0.254 |
| total SCFAs(μmol/L)**†** | 72.52(39.61,100.50) | 43.69(37.13,62.83) | 0.050 |

**†** Data are expressed as median(p25th-p75th); ‡ Data are expressed as mean±standard error.
